# Mental Health Symptoms of University Students 15 Months After the Onset of the COVID-19 Pandemic in France

**DOI:** 10.1001/jamanetworkopen.2022.49342

**Published:** 2022-12-29

**Authors:** Marielle Wathelet, Mathilde Horn, Coralie Creupelandt, Thomas Fovet, Thierry Baubet, Enguerrand Habran, Niels Martignène, Guillaume Vaiva, Fabien D’Hondt

**Affiliations:** 1 Department of Psychiatry, Centre Hospitalo-Universitaire de Lille, Lille, France; 2 Fédération de Recherche en Psychiatrie et Santé Mentale des Hauts-de-France, Lille, France; 3 Centre National de Ressources et de Résilience Lille-Paris, Lille, France; 4 University Lille, Inserm, Centre Hospitalo-Universitaire de Lille, U1172–Lille Neuroscience & Cognition, Lille, France; 5 Assistance Publique–Hôpitaux de Paris, Avicenne Hospital, Department of Infant, Child and Adolescent Psychiatry, Sorbonne Paris Nord University, Centre de recherche en Epidémiologie et Santé des Populations, Bobigny, France; 6 Fonds Fédération Hospitalière de France Recherche et Innovation, Paris, France

## Abstract

**Importance:**

The Conséquences de la pandémie de COVID-19 sur la santé mentale des étudiants (COSAMe) survey was conducted among university students in France during the COVID-19 pandemic and found that although there was a slight decrease in anxiety, depression, and stress between the first lockdown (T1) and 1 month after it ended (T2), the prevalence of suicidal ideation had increased between these periods and 1 in 5 students had probable posttraumatic stress disorder (PTSD) at T2. These results emphasize the need to explore the long-term consequences of the COVID-19 pandemic.

**Objectives:**

To measure the prevalence of mental health symptoms among university students in France 15 months after the first lockdown (T3) and to identify factors associated with outcomes.

**Design, Setting, and Participants:**

This cross-sectional study reports data from the third measurement time of the repeated COSAMe survey, which took place from July 21 to August 31, 2021, through an online questionnaire sent to all French university students.

**Main Outcomes and Measures:**

The prevalence of suicidal thoughts, PTSD (PTSD Checklist for the *Diagnostic and Statistical Manual of Mental Disorders* [Fifth Edition] [PCL-5]), stress (Perceived Stress Scale), anxiety (State-Trait Anxiety Inventory), and depression (Beck Depression Inventory) at T3 were gender- and degree-standardized and compared with prevalence rates at T1 and T2. Multivariable logistic regression analyses identified risk factors.

**Results:**

A total of 44 898 students completed the questionnaires. They were mainly women (31 728 [70.7%]), and the median (IQR) age was 19 (18-21) years. Standardized prevalence rates of stress, anxiety, depression, suicidal thoughts, and PTSD were 20.6% (95% CI, 20.2%-21.0%), 23.7% (95% CI, 23.3%-24.1%), 15.4% (95% CI, 15.1%-15.8%), 13.8% (95% CI, 13.5%-14.2%), and 29.8% (95% CI, 29.4%-30.2%), respectively. Compared with the decreased prevalence rates at T2, there was an increase at T3 for stress (2.5% increase), anxiety (13.9% increase), and depression (22.2% increase). The prevalence of suicidal ideation continued to increase from T1 (10.6%) to T3 (13.8%), and the prevalence of probable PTSD increased from 1 in 5 students to 1 in 3 students between T2 and T3. Female and nonbinary participants; participants without children and living in an urban area; and those with financial difficulties, a chronic condition, psychiatric history, COVID-19 history, social isolation, and low perceived quality of information received were at risk of all poor outcomes at T3 (eg, stress among women: adjusted OR, 2.18; 95% CI, 2.05-2.31; suicidal thoughts among nonbinary respondents: adjusted OR, 5.09; 95% CI, 4.32-5.99; anxiety among students with children: adjusted OR, 0.68; 95% CI, 0.56-0.81; depression among students living in a rural area: adjusted OR, 0.80; 95% CI, 0.75-0.85).

**Conclusions and Relevance:**

These results suggest severe long-lasting consequences associated with the pandemic on the mental health of students. Prevention and care access should be a priority.

## Key Points

**Question:**

Has the mental health of university students in France changed 15 months after the start of the COVID-19 pandemic?

**Findings:**

In this cross-sectional study of 44 898 university students who participated in the third measurement time of the Conséquences de la pandémie de COVID-19 sur la santé mentale des étudiants (COSAMe) survey, high prevalence rates for stress (20.6%), anxiety (23.7%), depression (15.4%), suicidal thoughts (13.8%), and posttraumatic stress disorder (29.8%) were observed.

**Meaning:**

These results suggest that the pandemic may have had long-lasting consequences on the mental health of students.

## Teaser

This cross-sectional study measures the prevalence of mental health symptoms among university students in France 15 months after the first COVID-19 lockdown and identifies factors associated with worse outcomes.

## Introduction

The COVID-19 pandemic had a major impact on mental health. Numerous studies conducted during the first months of the pandemic found high rates of mental health symptoms (stress, distress, anxiety, depression, posttraumatic stress) in the general population.^1^ The student population, whose vulnerability to mental health disorders was already well known,^2^ was quickly identified as particularly at risk of negative psychological repercussions from the pandemic and associated health measures.^1,3,4^

In France, the repeated cross-sectional survey Conséquences de la pandémie de COVID-19 sur la santé mentale des étudiants (COSAMe), whose first measurement time (T1) took place during the first lockdown (March 17 to May 11, 2020), reported high prevalence rates of severe self-reported stress (24.7%; 95% CI, 24.4%-25.1%), anxiety (27.5%; 95% CI, 27.1%-27.8%), depression (16.1%; 95% CI, 15.8%-16.4%), and suicidal thoughts (11.4%; 95% CI, 11.2%-11.7%) among the 69 054 participants. Overall, nearly half of students were affected by at least 1 severe mental health issue.^4^ During the second measurement period (T2), 1 month after the lifting of the lockdown, the prevalence of anxiety, depression, and stress had decreased without reaching prepandemic levels. In contrast, suicidal ideation increased from 11.4% to 13.2% (95% CI, 12.8%-13.6%), and symptoms of posttraumatic stress disorder (PTSD) were reported by nearly 1 in 5 students.^5^

The COVID-19 pandemic has been characterized by the occurrence of multiple waves of outbreaks and multiple measures deployed to limit the consequences of these waves.^6^ While there is still a growing body of research on the consequences of the pandemic on students’ mental health, studies assessing the long-term impacts are rarer. However, the direct (infections, hospitalizations, deaths) and indirect (economic crisis, difficulties in accessing care, isolation) consequences of the pandemic are likely to induce a very long-lasting mental health crisis,^7,8^ and recommendations invite monitoring the mental health of populations over the next few years.^9^ The present study used data from the third measurement time (T3) of the COSAMe survey, conducted 15 months after the beginning of the COVID-19 pandemic to (1) measure the prevalence rates of self-reported mental health symptoms (stress, anxiety, depression, PTSD, and suicidal thoughts) and (2) identify factors associated with mental health outcomes.

## Methods

### Study Design and Population

The study used data from the repeated cross-sectional university-based survey COSAMe, which consisted of 3 measurement times: T1, during the first lockdown (April 17 to May 4, 2020); T2, 1 month after the lift of the first lockdown (June 15 to July 15, 2020); and T3, 15 months after the start of the pandemic (July 21 to August 31, 2021). At each time, the French Ministry of Higher Education, Research, and Innovation requested the 82 universities to send an email to their students (target population, approximately 1 600 000 students) asking them to participate in the survey by completing online self-administered questionnaires. Due to the heterogeneity of sanitary measures from 1 country to another, the study only included students residing in France during the first lockdown.

The 2 first measurement times have already been analyzed, and the results have been published elsewhere.^4,5,10^ For this study, they are recalled to facilitate the interpretation of the third measurement time.

This survey was reviewed by a French research ethics committee, the Comité de Protection des Personnes Ile de France VIII, before its initiation. For T3, financial compensation was offered: €100 was awarded to 100 students randomly selected from those who completed the questionnaire entirely. To maintain anonymity, at the end of the questionnaire, students were directed to a page disconnected from the questionnaire, allowing them to enter their contact details to participate. Consent is not required for this type of observational study. An information note presented before the questionnaire informed the students about the study and the possibility of refusing to participate. Completion of the questionnaire was considered consent to participate. This study followed the Strengthening the Reporting of Observational Studies in Epidemiology (STROBE) reporting guideline.

### Collected Data

The following outcomes were screened: (1) suicidal thoughts, by asking participants whether they had experienced suicidal thoughts during the preceding month (yes or no); (2) PTSD, using the French version of the PTSD Checklist for *Diagnostic and Statistical Manual of Mental Disorders* (Fifth Edition) (PCL-5), a 20-item scale that explores PTSD symptom severity over the past month^11,12^; (3) stress, using the 10-item Perceived Stress Scale (PSS-10) to evaluate stress experiences during the preceding month^13,14,15^; (4) depression, using the 13-item Beck Depression Inventory (BDI-13) to assess current depression symptoms^16,17^; and (5) anxiety, using the 20-item State-Trait Anxiety Inventory, State subscale (STAI Y-2), to measure the intensity of current anxiety symptoms.^18,19^ The Cronbach α of the 4 scales in the sample were all greater than 0.89.

Outcomes were the presence of severe symptoms, ie, the presence of suicidal thoughts or a score above the threshold identified in the literature on 1 of the scales (PCL-5, >32 of 80; PSS-10, >26 of 40; BDI-13, >15 of 39; STAI Y-2, >55 of 80).^13,14,15,16,17,18,19,20,21^ We considered the following covariates to evaluate their association with the outcomes: (1) sociodemographic characteristics, age (in years), gender (male, female, other), academic degree (bachelor, master, doctorate), being a foreign student (yes, no), living area (urban, semiurban, rural), and having children (yes, no); (2) precariousness, financial difficulties (important for students reporting that it is difficult to make ends meet every month, moderate for participants for whom it is a bit difficult, low or no financial difficulties for others); (3) health-related data, history of psychiatric follow-up (benefiting from follow-up by a health professional for mental health reasons before the pandemic: yes, no), chronic condition (physical infirmity, handicap, or chronic disease: yes, no), COVID-19 (positive test for SARS-CoV-2 infection or suspected but without confirmatory test: yes, no); (4) social isolation, students who never physically meet or who only have very episodic contacts (sometimes in the year or less) with the members of all of their social networks (family, friends, neighbors, classmates, or members of their associative activities) were considered isolated; and (5) information data, perceived quality of information received about COVID-19 (on a scale of 10).

### Statistical Analysis

First, we described the sample using medians with IQRs for the scores of the measurement tools and quantitative covariates, since they were mostly not normally distributed. We used numbers and percentages for scores ranked by level and other qualitative variables.

To compare the results of T3 with those of T1 and T2, we calculated gender- and degree-standardized prevalence rates, using the university student population 2019 to 2020 published by the French Ministry of National Education^22^ and excluding nonbinary students given that their proportion among students was not available. We conducted χ^2^ tests to compare prevalence rates 2 by 2 (ie, T1 vs T2, T2 vs T3, T1 vs T3). PTSD was measured at T2 and T3 because according to the DSM-5, symptoms of PTSD must last for at least 1 month.^23^ At T1, we measured acute distress using the Impact of Events Scale–Revised.

Bivariate analyses were conducted to test the association between outcomes and covariates using χ^2^ tests, and multivariate logistic regression models identified risk factors of reporting at least 1 poor outcome (suicidal thoughts, PTSD, stress, depression, or anxiety) at T3. Then, similar models were conducted for each outcome. All explanatory variables were included except age due to collinearity with the year of study. Associations between risk factors and outcomes were presented as odds ratios (ORs) and 95% CIs.

Data analysis was performed using R version 3.6.1 (R Project for Statistical Computing). The level of significance was set at .05, and all tests were 2-sided.

## Results

### Sample Characteristics

A total of 55 457 students opened the online questionnaire. Among them, 44 898 completed (81.0%) it entirely and were analyzed.

The sample was mainly composed of women (31 728 [70.7%]), with 12 429 (27.7%) men and 741 students (1.6%) identifying as nonbinary. The median (IQR) age was 19 (18-21) years. Half of the respondents (22 716 [50.6%]) were in their first academic year, and 36 772 (81.9%) were bachelor students, whereas only 880 (2.0%) were in the sixth year or more. Among the participants, 3276 (7.3%) declared being foreign students, and 698 (1.5%) had children. Finally, 19 909 (44.3%) lived in an urban area, 11 808 (26.3%) in a semiurban area, and 13 181 (29.4%) in a rural area.

Nearly 1 in 8 students reported important financial difficulties (5830 [13.0%]). This proportion increased to 1 in 3 (15 844 [35.3%]) when we considered important and moderate financial difficulties together.

Regarding health information, 4002 respondents (8.9%) declared having a history of psychiatric follow-up, and 4713 (10.5%) reported having a chronic condition. More than one-quarter of the participants (12 105 [27.0%]) reported having had COVID-19 (positive test or suspicion without confirmatory test). Concerning social ties, 2013 (4.5%) were socially isolated. Finally, participants rated the quality of the information related to COVID-19 and quarantine, giving a median (IQR) score of 5 (3-7) of 10.

### Mental Health Outcomes and Associated Factors

Crude and standardized prevalence rates are described in Table 1. Among the 44 898 respondents, the crude prevalence rates of anxiety, depression, stress, PTSD, and suicidal thoughts were 25.0% (95% CI, 24.6%-25.4%), 16.9% (95% CI, 16.6%-17.3%), 22.5% (95% CI, 22.1%-22.9%), 31.0% (95% CI, 30.6%-31.4%), and 15.0% (14.7%-15.3%), respectively. After gender- and degree-standardization, prevalence rates were slightly lower (23.7% [95% CI, 23.3%-24.1%], 15.4% [95% CI, 15.1%-15.8%], 20.6% [95% CI, 20.2%-21.0%], 29.8% [95% CI, 29.4%-30.2%], and 13.8% [95% CI, 13.5%-14.2%], respectively). The medians (IQRs) of the scores were 45 (34-56) for the STAI Y-2 (anxiety), 8 (4-13) for the BDI-13 (depression), 20 (15-26) for the PSS-10 (stress), and 22 (10-37) for the PCL-5 (PTSD).

**Table 1.** Crude and Standardized Prevalence Rates of Mental Health Outcomes 15 Months After the Onset of the COVID-19 Pandemic Among the Whole Sample and According to Gender and Academic Degree

Characteristic	Participants, No. (%)						
	Characteristics		Mental health outcomes				
	Target population^a^	Study sample	Anxiety (STAI Y-2 score >55)	Depression (BDI score >15)	Stress (PSS-10 score >26)	PTSD (PCL-5 score >32)	Suicidal thoughts
Crude prevalence, % (95% CI)							
Including non-binary students	NA	44 898	25.0 (24.6-25.4)	16.9 (16.6-17.3)	22.5 (22.1-22.9)	31.0 (30.6-31.4)	15.0 (14.7-15.3)
Excluding non-binary students	NA	44 157	24.7 (24.3-25.1)	16.6 (16.2-16.9)	22.1 (21.7-22.5)	30.7 (30.3-31.1)	14.4 (14.1-14.7)
Standardized prevalence (95% CI)^b^	1 635 350	44 157	23.7 (23.3-24.1)	15.4 (15.1-15.8)	20.6 (20.2-21.0)	29.8 (29.4-30.2)	13.8 (13.5-14.2)
Bachelor							
Men	423 923 (25.9)	9880 (22.0)	1491 (15.1)	1197 (12.1)	1291 (13.1)	2262 (22.9)	1159 (11.7)
Women	573 542 (35.1)	26 267 (59.5)	7281 (27.7)	4917 (18.7)	6722 (25.6)	8763 (33.4)	4126 (15.7)
Master							
Men	234 829 (14.3)	2266 (5.4)	440 (19.4)	292 (12.9)	329 (14.5)	631 (27.8)	286 (12.6)
Women	347 872 (21.3)	4878 (14.5)	1491 (30.6)	797 (16.3)	1275 (26.1)	1669 (34.2)	719 (14.7)
Doctorate							
Men	28 365 (1.7)	283 (0.8)	58 (20.5)	33 (11.7)	36 (12.7)	70 (24.7)	26 (9.2)
Women	26 819 (1.6)	583 (1.9)	163 (28.0)	75 (12.9)	128 (22.0)	162 (27.8)	64 (11.0)

Abbreviations: BDI, Beck Depression Inventory; NA, not applicable; PCL-5, PTSD Checklist for DSM-5; PSS-10, 10-item Perceived Stress Scale; STAI Y-2, State-Trait Anxiety Inventory, State subscale.

^a^ University students population from 2019 to 2020.

^b^ Gender- and degree-standardized prevalence rates.

The Figure presents the standardized prevalence rates measured at T3 as well as those measured previously at T2 and T1. For stress, depression, and anxiety, a V-shaped pattern was identified, with a decrease in prevalence at T2 and an increase at T3. Prevalence rates at T3 were lower than those at T1 for stress (20.6% vs 22.4%) and anxiety (23.7% vs 25.5%), but higher for depression (15.4% vs 14.3%); however, these results represented a 2.5% increase for stress, a 13.8% for anxiety, and a 22.2% increase for depression compared with T2. The prevalence of PTSD was particularly high at T3, with 29.8% of students affected, compared with 18.4% at T2. Finally, the prevalence of suicidal ideation increased since the beginning of the survey, reaching 13.8% at T3, against 12.3% at T2 and 10.6% at T1. All tests performed were significant (*P* < .001), except for the comparison of stress prevalence between T2 and T3 (*P* = .13).

**Figure.**
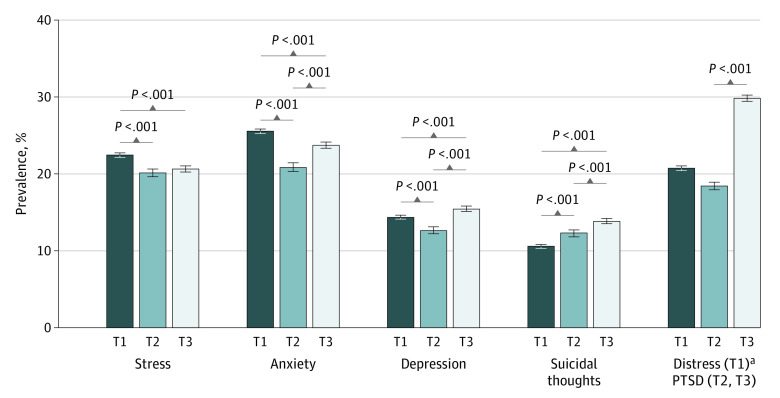
Standardized Prevalence Rates of Mental Health Disorders During the First Lockdown (T1), 1 Month after the First Lockdown Ended (T2), and 15 Months After the Onset of the COVID-19 Pandemic (T3) Standardized prevalence rates for participants, excluding nonbinary students, were calculated, creating a sample size of 68 106 students for T1; 22 205, T2; and 44 157, T3. Stress decreased 10.3% between T1 and T2 and increased 2.5% between T2 and T3. Anxiety decreased 18.4% between T1 and T2 and increased 13.9% between T2 and T3. Depression decreased 11.9% between T1 and T2 and increased 22.2% between T2 and T3. Suicidal thoughts increased 16.0% between T1 and T2 and 12.2% between T2 and T3. Posttraumatic stress disorder (PTSD) increased 61.9% between T2 and T3. ^a^Comparisons between T3 and T1 and between T2 and T1 were not conducted because acute distress was measured at T1 (using the Impact of Events Scale–Revised) rather than PTSD.

### Factors Associated With Mental Health Outcomes

Bivariate analyses are presented in Table 2. Multivariate analyses are presented in Table 3.

**Table 2.** Factors Associated With Poor Mental Health Outcomes 15 Months After the Onset of the COVID-19 Pandemic According to the Bivariate Analysis

Factor	No. (%) (N = 44 898)	Stress (PSS-10 score >26)		Anxiety (STAI Y-2 score >55)		Depression (BDI-13 score >15)		PTSD (PCL-5 score >32)		Suicidal thoughts	
		No. (%)	*P* value	No. (%)	*P* value	No. (%)	*P* value	No. (%)	*P* value	No. (%)	*P* value
Gender											
Male	12 429 (27.7)	1656 (13.3)	<.001	1989 (16.0)	<.001	1522 (12.2)	<.001	2963 (23.8)	<.001	1471 (11.8)	<.001
Female	31 728 (70.7)	8125 (25.6)		8905 (28.1)		5789 (18.2)		10 594 (33.4)		4909 (15.5)	
Other	741 (1.6)	340 (45.9)		350 (47.2)		292 (39.4)		358 (48.3)		352 (47.5)	
Degree											
Bachelor	36 772 (81.9)	8306 (22.6)	.03	9069 (24.7)	<.001	6367 (17.3)	<.001	11 327 (30.8)	<.001	5592 (15.2)	<.001
Master	7246 (16.1)	1649 (22.7)		1949 (26.9)		1127 (15.5)		2351 (32.4)		1045 (14.4)	
Doctorate	880 (2.0)	166 (18.9)		226 (25.7)		109 (12.4)		237 (26.9)		95 (10.8)	
Foreign student											
Yes	3276 (7.3)	692 (21.1)	.046	920 (28.1)	<.001	642 (19.6)	<.001	1440 (43.9)	<.001	481 (14.7)	.62
No	41 622 (92.7)	9429 (22.6)		10 324 (24.8)		6961 (16.7)		12 475 (30.0)		6251 (15.0)	
Having children											
Yes	698 (1.5)	124 (17.8)	.003	167 (23.9)	.52	99 (14.2)	.06	231 (33.1)	.24	80 (11.5)	.01
No	44 200 (98.5)	9997 (22.6)		11 077 (25.1)		7504 (17.0)		13 684 (31.0)		6652 (15.0)	
Living area											
Urban	19 909 (44.3)	4650 (23.3)	<.001	5175 (26.0)	<.001	3660 (18.4)	<.001	6507 (32.7)	<.001	3113 (15.6)	<.001
Semiurban	11 808 (26.3)	2728 (23.1)		2987 (25.3)		2019 (17.1)		3626 (30.7)		1846 (15.6)	
Rural	13 181 (29.4)	2743 (20.8)		3082 (23.4)		1924 (14.6)		3782 (28.7)		1773 (13.4)	
Financial difficulties											
No or few	29 054 (64.7)	5140 (17.7)	<.001	5602 (19.3)	<.001	3556 (12.2)	<.001	7118 (24.5)	<.001	3590 (12.3)	<.001
Moderate	10 014 (22.3)	2715 (27.1)		3067 (30.6)		2061 (20.6)		3826 (38.2)		1693 (16.9)	
Important	5830 (13.0)	2266 (38.9)		2575 (44.2)		1986 (34.1)		2971 (51.0)		1449 (24.8)	
History of psychiatric follow-up											
Yes	4002 (8.9)	1632 (40.8)	<.001	1738 (43.4)	<.001	1362 (34.0)	<.001	1968 (49.2)	<.001	1348 (33.7)	<.001
No	40 896 (91.1)	8489 (20.7)		9506 (23.2)		6241 (15.3)		11 947 (29.2)		5384 (13.2)	
Chronic condition											
Yes	4713 (10.5)	1591 (33.7)	<.001	1728 (36.7)	<.001	1268 (26.9)	<.001	2023 (42.9)	<.001	1184 (25.1)	<.001
No	40 185 (89.5)	8530 (21.2)		9516 (23.7)		6335 (15.8)		11 892 (29.6)		5548 (13.8)	
COVID-19 (suspected or positive test)											
Yes	12 105 (27.0)	3064 (25.3)	<.001	3390 (28.0)	<.001	2282 (18.8)	<.001	4495 (37.1)	<.001	1995 (16.5)	<.001
No	32 793 (73.0)	7057 (21.5)		7854 (23.9)		5321 (16.2)		9420 (28.7)		4737 (14.4)	
Social isolation											
Yes	2013 (4.5)	650 (32.3)	<.001	735 (36.5)	<.001	593 (29.4)	<.001	745 (37.0)	<.001	522 (25.9)	<.001
No	42 885 (95.5)	9471 (22.1)		10 509 (24.5)		7010 (16.3)		13 170 (30.7)		6210 (14.5)	
Quality of information received											
High (7-10)	12 837 (28.6)	2281 (17.8)	<.001	2529 (22.1)	<.001	1692 (14.8)	<.001	3275 (28.6)	<.001	1607 (12.5)	<.001
Medium (4-6)	20 268 (45.9)	4562 (22.5)		5141 (25.4)		3433 (16.9)		6240 (30.8)		2987 (14.7)	
Low (0-3)	11 433 (25.5)	3278 (28.7)		3574 (27.8)		2478 (19.3)		4400 (34.3)		2138 (18.7)	

Abbreviations: BDI-13, 13-item Beck Depression Inventory; PCL-5, Posttraumatic Stress Disorder Checklist for DSM-5; PTSD, posttraumatic stress disorder; PSS-10, 10-item Perceived Stress Scale; STAI Y-2, 20-item State-Trait Anxiety Inventory, State subscale.

**Table 3.** Factors Associated With Poor Mental Health Outcomes 15 Months After the Onset of the COVID-19 Pandemic According to the Multivariate Logistic Regression Analysis

Factor	Stress (PSS-10 >26)		Anxiety (STAI Y-2 >55)		Depression (BDI-13 >15)		PTSD (PCL-5 >32)		Suicidal thoughts	
	Adjusted OR (95% CI)	*P* value	Adjusted OR (95% CI)	*P* value	Adjusted OR (95% CI)	*P* value	Adjusted OR (95% CI)	*P* value	Adjusted OR (95% CI)	*P* value
Gender										
Male	1 [Reference]	<.001	1 [Reference]	<.001	1 [Reference]	<.001	1 [Reference]	<.001	1 [Reference]	<.001
Female	2.18 (2.05-2.31)		2.01 (1.90-2.13)		1.53 (1.43-1.63)		1.60 (1.52-1.68)		1.28 (1.20-1.36)	
Other	4.34 (3.69-5.10)		3.79 (3.23-4.45)		3.58 (3.02-4.23)		2.50 (2.13-2.93)		5.09 (4.32-5.99)	
Degree										
Bachelor	1 [Reference]	.13	1 [Reference]	.001	1 [Reference]	<.001	1 [Reference]	<.001	1 [Reference]	<.001
Master	1.02 (0.96-1.09)		1.12 (1.05-1.19)		0.82 (0.76-0.88)		1.00 (0.94-1.06)		0.91 (0.84-0.98)	
Doctorate	0.84 (0.70-1.01)		1.10 (0.93-1.29)		0.63 (0.51-0.78)		0.74 (0.63-0.87)		0.64 (0.51-0.80)	
Foreign student (yes vs no)	0.91 (0.83-1.00)	.05	1.14 (1.04-1.24)	.003	1.18 (1.07-1.31)	.001	1.87 (1.72-2.02)	<.001	1.00 (0.90-1.11)	.99
Having children (yes vs no)	0.56 (0.45-0.68)	<.001	0.68 (0.56-0.81)	<.001	0.61 (0.49-0.76)	<.001	0.82 (0.69-0.97)	.02	0.61 (0.47-0.77)	<.001
Living area										
Urban	1 [Reference]	<.001	1 [Reference]	.001	1 [Reference]	<.001	1 [Reference]	<.001	1 [Reference]	<.001
Semiurban	1.02 (0.97-1.08)		1.03 (0.97-1.09)		0.97 (0.91-1.03)		0.99 (0.94-1.04)		1.04 (0.97-1.11)	
Rural	0.88 (0.84-0.93)		0.92 (0.88-0.98)		0.80 (0.75-0.85)		0.91 (0.86-0.95)		0.88 (0.82-0.94)	
Financial difficulties										
No or few	1 [Reference]	<.001	1 [Reference]	<.001	1 [Reference]	<.001	1 [Reference]	<.001	1 [Reference]	<.001
Moderate	1.62 (1.53-1.71)		1.73 (1.64-1.82)		1.73 (1.63-1.84)		1.75 (1.67-1.84)		1.36 (1.27-1.45)	
Important	2.81 (2.63-2.99)		3.10 (2.91-3.30)		3.44 (3.21-3.68)		2.82 (2.65-2.99)		2.19 (2.03-2.35)	
History of psychiatric follow-up (yes vs no)	2.19 (2.04-2.35)	<.001	2.14 (1.99-2.30)	<.001	2.45 (2.27-2.64)	<.001	2.06 (1.92-2.21)	<.001	2.81 (2.60-3.03)	<.001
Chronic condition (yes vs no)	1.53 (1.42-1.64)	<.001	1.51 (1.41-1.62)	<.001	1.53 (1.42-1.65)	<.001	1.49 (1.39-1.59)	<.001	1.62 (1.50-1.75)	<.001
Suspected or confirmed COVID-19 (yes vs no)	1.18 (1.12-1.24)	<.001	1.18 (1.12-1.24)	<.001	1.13 (1.07-1.20)	<.001	1.42 (1.36-1.49)	<.001	1.12 (1.05-1.19)	<.001
Social isolation (yes vs no)	1.63 (1.47-1.81)	<.001	1.73 (1.56-1.91)	<.001	2.07 (1.86-2.30)	<.001	1.28 (1.16-1.41)	<.001	1.96 (1.76-2.18)	<.001
Quality of information received										
High (7-10)	1 [Reference]	<.001	1 [Reference]	<.001	1 [Reference]	<.001	1 [Reference]	<.001	1 [Reference]	<.001
Medium (4-6)	1.25 (1.18-1.32)		1.30 (1.23-1.38)		1.28 (1.20-1.37)		1.25 (1.19-1.32)		1.36 (1.30-1.42)	
Low (0-3)	1.74 (1.63-1.85)		1.76 (1.65-1.87)		1.70 (1.58-1.82)		1.80 (1.70-1.91)		1.57 (1.48-1.66)	

Abbreviations: BDI-13, 13-item Beck Depression Inventory; OR, odds ratio; PCL-5, Posttraumatic Stress Disorder Checklist for DSM-5; PTSD, posttraumatic stress disorder; PSS-10, 10-item Perceived Stress Scale; STAI Y-2, 20-item State-Trait Anxiety Inventory, State subscale.

#### Sociodemographic Characteristics

After adjustment, women and nonbinary students had increased risks of poor mental health symptoms compared with men (eg, stress among women: adjusted OR, 2.18; 95% CI, 2.05-2.31; suicidal thoughts among nonbinary respondents: adjusted OR, 5.09; 95% CI, 4.32-5.99). On the contrary, having children and living in a rural area (vs urban area) were associated with less risk (eg, anxiety among students with children: adjusted OR, 0.68; 95% CI, 0.56-0.81; depression among students living in a rural area: adjusted OR, 0.80; 95% CI, 0.75-0.85). Academic degree program was associated with all outcomes, except stress, but with less clear patterns. Compared with bachelor students, PhD students had lower risk of depression (OR, 0.63; 95% CI, 0.51-0.78; *P* < .001), PTSD (OR, 0.74; 95% CI, 0.63-0.87; *P* < .001), and suicidal thoughts (OR, 0.64; 95% CI, 0.51-0.80; *P* < .001). Master students were less at risk for depression (OR, 0.82; 95% CI, 0.76-0.88; *P* < .001) and suicidal thoughts (OR, 0.91; 95% CI, 0.84-0.98; *P* = .02) but at higher risk for anxiety (OR, 1.12; 95% CI, 1.05-1.19; *P* < .001). Being a foreign student was associated with a higher risk of depression (OR, 1.14; 95% CI, 1.04-1.24; *P* = .003), PTSD (OR, 1.18; 95% CI, 1.07-1.31; *P* = .001), and suicidal thoughts (OR, 1.87; 95% CI, 1.72-2.02; *P* < .001).

#### Financial Difficulties

For all mental health outcomes, the greater the financial difficulties reported by the students, the higher the risk of a poor outcome. Compared with students with no or few difficulties, those reporting moderate difficulties had ORs ranging from 1.36 (95% CI, 1.27-1.45) for suicidal thoughts to 1.75 (95% CI, 1.67-1.84) for PTSD, and those declaring significant difficulties had ORs ranging from 2.19 (95% CI, 2.03-2.35) for suicidal thoughts to 3.44 (95% CI, 3.21-3.68) for depression.

#### Health-Related Data

Prevalence rates of mental health disorders were significantly higher among students with a history of COVID-19 (confirmed or suspected), with a chronic condition, and with a history of psychiatry follow-up. ORs ranged from 1.12 (95% CI, 1.05-1.19) for suicidal thoughts to 1.42 (95% CI, 1.36-1.49) for PTSD among those with a history of COVID-19, from 1.49 (95% CI, 1.39-1.59) for PTSD to 1.62 (95% CI, 1.50-1.75) for suicidal thoughts among those with a chronic condition, and from 2.06 (95% CI, 1.92-2.21) for PTSD to 2.81 (95% CI, 2.60-3.03) for suicidal thoughts among those with psychiatry history.

#### Social Isolation

Socially isolated students were consistently at higher risk for mental health issues. ORs ranged from 1.28 (95% CI, 1.16-1.41; *P* < .001) for PTSD to 2.07 (95% CI, 1.86-2.30; *P* < .001) for depression.

#### Information and Media

The lower the quality of the information received, the more the students were at risk for severe mental health issues. ORs ranged from 1.25 (95% CI, 1.19-1.32) for PTSD to 1.36 (95% CI, 1.30-1.42) for suicidal thoughts if they rated the quality with a score between 4 and 5 compared with a score greater than 6, and from 1.57 (95% CI, 1.48-1.66) for suicidal thoughts to 1.80 (95% CI, 1.70-1.91) for PTSD if they rated the quality with a score less than 4.

## Discussion

This large nationwide study found high rates of stress, anxiety, depression, suicidal thoughts, and PTSD among university students in France 15 months after the start of the pandemic. By comparing these results with the 2 previous measurement times of the COSAMe survey (during the first lockdown and 1 month after it ended), a V-shaped pattern was observed for anxiety and depression, ie, an increase following a drop in the prevalence rates observed after the lifting of the first lockdown. Only the prevalence of suicidal thoughts has been steadily increasing since the first lockdown. The prevalence of PTSD has reached important levels 15 months after the beginning of the pandemic, with nearly 1 in 3 students concerned. Women and nonbinary students, those without children and living in an urban area, and those with financial difficulties, social isolation, history of psychiatric follow-up, history of COVID-19 (suspected or confirmed), a chronic condition, and low assessment of the quality of the information received had increased risk of mental health issues.

Comparisons with other studies are complex insofar as the prevalence of disorders is influenced by health restrictions (which differed from one country to another), study period (given variations in restrictions over time and seasonality of certain mental disorders), the type of population concerned (as vulnerabilities may vary), and the measuring tools (whose psychometric properties may differ).^24^ However, our results are consistent with the study by Charbonnier et al,^24^ who measured levels of anxiety and depression among French university students. Using the Hospital Anxiety and Depression Scale, the authors found a higher prevalence of probable depression (between 12.1% and 26.4%) and anxiety (between 26.6% and 45.0%) in 2021 compared with 2020.^24^ Results are also consistent with the study by Schmits et al,^28^ including 23 307 French university students 1 year after the beginning of the pandemic. The authors reported that 50.6% of participants described anxiety symptoms, 55.1% described depressive symptoms, and 20.8% described suicidal ideation.^28^ However, this study was cross-sectional without a prior point of comparison. A few longitudinal studies have been conducted in the general population over a period like that of our study. A study of 1838 Belgian adults, conducted from April 2020 to June 2021,^25^ showed that the prevalence of symptoms of anxiety and depression was higher in times of stricter policy measures. This study did not include student status. However, although time trends were similar for all population subgroups, higher levels of both anxiety and depression were generally found in young people.^25^ Conversely, a longitudinal study conducted among 988 adults in Argentina showed a gradual increase in anxiety and depression between August 2020 and April 2021.^26^ Again, young adults had higher prevalence rates of symptoms than other age groups.^26^ Finally, in the study by Lu et al,^27^ including 613 French adults, a continuing increase in the mean scores of anxiety and depression symptoms was observed throughout the 2 lockdown periods in France, with younger participants being more vulnerable to anxiety symptoms.

The risk factors identified in our study are similar to those identified in the previous measurement times of the COSAMe survey and consistent with those described in the literature on pandemics or lockdowns. The review by Brooks et al^3^ pointed out that gender, psychiatric history, physical symptoms, social isolation, lack of information, and financial loss were all associated with mental health conditions. Schmits et al^28^ also identified women and nonbinary individuals as having increased risk for poor mental health as well as a deterioration of the financial situation and reduced contact with family and friends. Of note, for most students, the academic year at T3 differed from the academic year at the time of exposure. Except for students repeating a year or returning to university after a break, they all had been confined while they were in the lower academic year, including the last high school year for first-year students at T3. Consequently, standardization on the current academic degree does not correspond to standardization on the academic degree at the time of exposure, and this may slightly bias the comparisons of the results at T3 with those at T1 and T2. Nevertheless, whatever the measurement time, we found that the prevalence of mental health disorders tended to decrease among those pursuing a higher degree, and in particular, that the risk appeared to be lower among doctoral students. This association is consistent with the literature that has established a strong link between mental health and the level of education.^29^

Even if the situation were still unstable in the summer of 2021, restrictions had been reduced compared with the strict lockdown periods. This improvement in the COVID-19 health crisis was instead accompanied by a worsening of the mental health of students. Several hypotheses can explain this situation. The first explanation is that the health crisis has led to a social and economic crisis, with well-known consequences.^30^ It has already been shown that, during periods of crisis (natural disasters, war, or epidemics), suicide rates may momentarily decrease before increasing thereafter, particularly under the influence of economic repercussions and social of the crisis.^8,31^ The occurrence or increase of unemployment, poverty, or even loneliness is likely to contribute to the increase in mental health disorders, such as depression, anxiety, and PTSD, and the suicide rate.^8^ However, the specific evolution in the prevalence of PTSD, which reached a particularly high level at T3, raises questions. The recent network approach to psychopathology explains the persistence of disorders over time, even after the disappearance of the initial triggering event (eg, the first lockdown).^32^ It posits that mental disorders can be conceptualized and studied as causal systems of mutually reinforcing symptoms^33^: according to this theory, there are causal associations between mental health symptoms, and if these associations are strong enough, the symptoms can be self-perpetuating, regardless of the event that initially triggered them.^34^ The COVID-19 pandemic is an unprecedented and particularly complex event, which may be better understood as a collection of several events that are direct or indirect consequences of COVID-19. We hypothesize that the COVID-19 pandemic and its related consequences are underestimated and that prolonged and cumulative exposure to stressors and/or potentially traumatic events during this period could have led to the occurrence or decompensation of mental health disorders, self-sustaining even after the health situation has improved. Although recent and still unstable, exploring the network approach to mental disorders could help to better understand mental health symptoms interact with each other, contributing to a better understanding of how mental disorders persist over time.

### Limitations

This study has limitations. First, although the number of respondents is large, it represents a minority of students (2.8%). The overrepresentation of women and bachelor students was considered by standardizing on gender and academic degree, the only 2 variables common to our sample and those available at the national level via the Ministry of Education. Association measures are only marginally affected by a low response rate. Second, self-administrated tools used in this study cannot be considered diagnostic tools. Third, the present study cannot establish the direct link between the high rates of mental health disorders and the COVID-19 pandemic and its associated restriction measures, even though high rates were also observed in other studies related to the COVID-19 pandemic or previous pandemics.^3,35^ Fourth, this survey did not include any measures prior to the pandemic. However, as discussed by Wathelet et al^4^ regarding estimates obtained at T1, the prevalence rates measured were higher than the prepandemic measurements identified in the literature. Fifth, the seasonality of certain mental disorders might be a subject of concern. Indeed, in our study, T3, unlike T2 and T1, was conducted exclusively during the summer holidays. Although the phenomenon is complex and poorly understood and contrary to most mental health disorders,^36^ an increased risk of suicide during late spring and early summer has been observed.^37^ Among students, the differences in suicidal ideation between summer and winter were shown to be, in large part, accounted for by belongingness.^38^ That being said, although seasonality cannot be strictly controlled here, all of the measurement times took place during spring or summer periods, which limits the bias in the comparisons. Additionally, some other factors could be associated with mental health disorders but have not been considered here, such as relationship, residence, or institution type.

## Conclusions

This large nationwide study found high prevalence rates of anxiety, depression, perceived stress, PTSD, and suicidal ideation 15 months after the beginning of the COVID-19 pandemic among university students in France. If a slight decrease had been observed just after the first lockdown for anxiety and depression, evidence shows that suicidal ideation has increased throughout the survey and that PTSD has jumped from 1 in 5 to 1 in 3 students concerned. These results suggest long-lasting consequences associated with the pandemic on the mental health of students. Prevention and care access should be a priority.

## Notes

Supplement.
Data Sharing Statement
