# Supplementary material for: Mental Health Symptoms of University Students 15 Months After the Onset of the COVID-19 Pandemic in France
Source: JAMA Netw Open. 2022 Dec 29;5(12):e2249342. doi: 10.1001/jamanetworkopen.2022.49342 (PMC9857035; doi:10.1001/jamanetworkopen.2022.49342)
Supplement: Supplement. — Data Sharing Statement [file jamanetwopen-e2249342-s001.pdf]

Data Sharing Statement

Wathelet. Mental Health Symptoms of University Students 15 Months After the Onset of the COVID-19 Pandemic in France. *JAMA Netw Open*. Published December 29, 2022. doi:10.1001/jamanetworkopen.2022.49342

Data
Data available: Yes
Data types: Deidentified participant data, Data dictionary
How to access data: Data will be made avaibable upon request to the corresponding author: watheletmarielle@gmail.com
When available: With publication

Supporting Documents
Document types: None

Additional Information
Who can access the data: researchers whose proposed use of the data has been approved
Types of analyses: researchers whose proposed use of the data has been approved
Mechanisms of data availability: after approval of a proposal
